# Genotypic and Phenotypic Detection of Fosfomycin and Tigecycline Resistance Genes From Clinical Isolates of Enterobacter cloacae

**DOI:** 10.7759/cureus.101259

**Published:** 2026-01-10

**Authors:** Sanjida Hossain, Shahnaz Choudhury, AKM Mohiuddin Bhuiyan, Adneen Moureen, Redoy Ranjan, SK Jakaria Been Sayeed, Abdullah Yusuf

**Affiliations:** 1 Department of Microbiology, Dr. Sirajul Islam Medical College, Dhaka, BGD; 2 Department of Radiology and Imaging, Bangladesh Medical University, Dhaka, BGD; 3 Department of Cardiology, Popular Medical College & Hospital, Dhaka, BGD; 4 Department of Microbiology, Armed Forces Medical College, Dhaka, BGD; 5 Department of Cardiac Surgery, St Georges University Hospital NHS Foundation Trust, London, GBR; 6 Department of Cardiac Surgery, Bangladesh Medical University, Dhaka, BGD; 7 Department of Biological Science, Royal Holloway University of London, London, GBR; 8 Department of Neurology, National Institute of Neurosciences Hospital, Dhaka, BGD; 9 Department of Microbiology, National Institute of Neurosciences Hospital, Dhaka, BGD

**Keywords:** agar dilution method, antibiotic resistance genes, bacteriuria fosfomycin, enterobacter cloacae, multidrug resistant (mdr), polymerase chain reaction, tigecycline

## Abstract

Background

The emergence of multidrug-resistant (MDR) *Enterobacter* species is a growing concern, driving increased morbidity and mortality, particularly due to hospital-acquired infections.

Objective

This study aimed to identify multidrug-resistant *Enterobacter cloacae* through the detection of antibiotic resistance genes.

Methodology

This cross-sectional study was conducted in the Department of Microbiology at Dhaka Medical College, Dhaka, Bangladesh, from November 2022 to June 2023 for a period of one year. All the clinically suspected patients who were presented with the infection of different body sites were divided into two groups: male and female. Then, each group is segmented into four specific age brackets: 20-30, 31-40, 41-50, and 51-60. The multidrug-resistant *Enterobacter cloacae* were isolated from patients admitted to Dhaka Medical College Hospital, Dhaka, Bangladesh. Samples, including urine, wound swabs, endotracheal aspirates, and blood, were collected from Dhaka Medical College's microbiology lab. *Enterobacter* species were identified through culture, Gram staining, and biochemical tests. Antibiotic susceptibility was tested using the disc diffusion method. PCR detected fosfomycin (*fosA, fosA3, fosA4, fosB*, *fosC*, *fosX*, *fosL1)* and tigecycline (*tet*X4, *tetA*) resistance genes.

Results

Among 390 samples, 251 (64.4%) isolates were culture-positive, of which 35 (13.9%) isolates were identified as *Enterobacter* species. Biochemical tests revealed 30 (85.7%) *Enterobacter cloacae* and five (14.3%) *Enterobacter aerogenes*. Among the isolated *Enterobacter* species, 19 (56.7%) isolates were MDR, five (16.7%) isolates were extensively drug-resistant (XDR), and three (10.0%) isolates were pan drug-resistant (PDR) *Enterobacter cloacae* isolates. Among 30 *Enterobacter cloacae* isolates, 29 (96.7%) were resistant to ceftriaxone, while 11 (36.7%) had the lowest resistance to tigecycline. Fosfomycin and imipenem resistance were observed in 22 (66.7%) and 21 (70.0%) of isolates, respectively. Among 20 fosfomycin-resistant *Enterobacter cloacae*, 13 (65.0%) carried *fosA*, four (20.0%) *fosA3*, and three (15.0%) *fosA4*. Among 11 tigecycline-resistant *Enterobacter cloacae*, *tetA* was detected in three (27.3%).

Conclusion

In conclusion, *Enterobacter* species often exhibit strong resistance to various antibiotics, performing antimicrobial susceptibility testing is essential before initiating treatment.

## Introduction

*Enterobacter* species are widely present in nature and often inhabit the intestinal microbiota of both humans and animals, with the capacity to survive on skin and dry surfaces, as well as to proliferate in contaminated liquids [1-2]. *Enterobacter* species are significant contributors to hospital-acquired infections and are frequently isolated [3]. Among the *Enterobacter *genus, *Enterobacter cloacae* and *Enterobacter aerogenes* are the species most commonly recovered [4]. The *Enterobacter *species mainly cause nosocomial infections, including UTIs, respiratory infections, soft tissue infections, osteomyelitis, and endocarditis [5]. In the United States, *Enterobacter *spp. ranks as the second most prevalent carbapenem-resistant Enterobacteriaceae (CRE) [6].

The rising antimicrobial resistance of *Enterobacter cloacae* is a significant concern. It has particularly given over 60.0% resistance to critical antibiotics such as polymyxin B, tigecycline, and nitrofurantoin. Despite ongoing efforts to develop new antibiotics for treating these resistant infections, progress has been minimal. This situation necessitates coordinated efforts at multiple levels to improve the management of AMR infections [7]. While most cases are linked to healthcare environments, instances in community settings are on the rise. Alterations in porin channels, the presence of integrons and insertion sequence common region 1 (ISCR1) carrying resistance genes, along with the activity of efflux pumps, collectively play a role in conferring these resistance traits [8].

The rise of carbapenem non-susceptible strains is largely due to the production of carbapenemases, as well as extended-spectrum beta-lactamases (ESBL) and AmpC beta-lactamases. The action of efflux pumps also contributes to antimicrobial resistance [9]. It's not unexpected that carbapenem-resistant *Enterobacter* species are classified as ESKAPE pathogens, which are deemed some of the most concerning healthcare-associated pathogens globally [10]. This study aimed to identify multidrug-resistant *Enterobacter cloacae* through the detection of antibiotic resistance genes.

## Materials and methods

Study settings and population

This cross-sectional study was conducted in the Department of Microbiology at Dhaka Medical College, Dhaka, Bangladesh, from November 2022 to June 2023 for a period of one year. All the clinically suspected patients who were presented with the infection of different body sites were divided into two groups: male and female. Then, each group is segmented into four specific age brackets: 20-30, 31-40, 41-50, and 51-60. The multidrug-resistant *Enterobacter cloacae *were isolated from patients admitted to Dhaka Medical College Hospital, Dhaka, Bangladesh. Dhaka Medical College Hospital, Dhaka, is situated in the capital of Bangladesh with a capacity of 1500 beds. However, there are more than 4000 patients who were admitted to this hospital. This is a public general teaching hospital. All the subjects related to the departments of medicine and surgery are available here. Bangladesh is a densely populated country with a population of 18 million. Patients from all corners of this country are admitted with different infectious diseases.

Selection criteria

In this study, the suspected cases of nosocomial infection were included, and the samples according to the site of infection were collected, which included urine, blood, wound swab, and pus samples from patients who were admitted to different indoor departments of Dhaka Medical College Hospital, Dhaka, Bangladesh. The endotracheal aspirates from ICU patients on mechanical ventilation for over 48 hours were also collected. Samples containing more than one organism were excluded from this study.

Study procedure

All samples were processed in the microbiology department for culture and sensitivity testing. Furthermore, the isolated bacteria were monitored for their antibiotic resistance pattern by the disk diffusion method. 

Sample collection and species identification

The study collected 35 Enterobacter isolates from various clinical samples, including urine, wound swabs, endotracheal aspirates, and blood. Two Enterobacter species, *Enterobacter cloacae* and *Enterobacter aerogenes*, were identified through a series of biochemical tests, which included the Lysine decarboxylase test. 

Antibiotic susceptibility testing

The antibiotic susceptibility of *Enterobacter cloacae* was evaluated using the Kirby-Bauer disc diffusion technique on Mueller-Hinton agar, with antibiotic discs obtained from Oxoid Ltd, UK. The antibiotics evaluated in this study were amikacin (30 µg), amoxiclav (30 µg), ceftazidime (30 µg), colistin (10 µg), ceftriaxone (30 µg), ciprofloxacin (30 µg), piperacillin/tazobactam (110/10 µg), imipenem (10 µg), tigecycline (15 µg), and gentamicin (30 µg). For the evaluation of tigecycline, imipenem, and fosfomycin susceptibility, provided by Beximco Pharma Limited, the agar dilution method was utilized to determine the minimum inhibitory concentration (MIC). The disc content and inhibition zones were interpreted according to the Clinical Laboratory Standards Institute guidelines (CLSI, 2022), while the interpretation of tigecycline results specifically followed criteria established by the United States Food and Drug Administration (2010). A 0.5 McFarland standard (equivalent to 1×10⁸ cfu/ml) was used for inoculum preparation and MIC testing, followed by a tenfold dilution to obtain a final concentration of 1×10⁷ cfu/ml. The inoculated plates were incubated aerobically at 37ºC overnight, and the MIC was recorded as the lowest antibiotic concentration on Mueller-Hinton agar that prevented visible bacterial growth. The definition and classification of multidrug-resistant (MDR), pan drug-resistant (PDR), and extensively drug-resistant (XDR) were based on the standard criteria of the CDC. *Escherichia coli *ATCC 25922 served as the quality control organism.

Polymerase chain reaction

To identify MDR genes in *Enterobacter *spp., polymerase chain reaction (PCR) was performed. A loopful of bacterial colonies from Mueller-Hinton agar (MHA) was collected and suspended in a microcentrifuge tube containing sterile tryptic soy broth (TSB), then incubated overnight at 37ºC. After incubation, the tube was centrifuged at 4000 g for 10 minutes, the supernatant discarded, and the bacterial pellet stored at -20ºC for subsequent DNA extraction. For DNA extraction, 300 µl of sterile distilled water was added to the pellet, vortexed thoroughly, and heated at 100ºC for 10 minutes. The tubes were then cooled on ice for five minutes and centrifuged at 14,000 g for six minutes at 4ºC, after which the supernatant containing DNA was transferred to a new tube. Extracted DNA was stored at 4ºC for 7-10 days or at -20ºC for long-term use. For PCR, a 25 µl reaction mixture was prepared in a PCR tube, containing 12.5 µl of mastermix (with dNTPs, Taq polymerase, MgCl₂, and PCR buffer), 2 µl each of forward and reverse primers (Promega Corporation, USA), 2 µl of extracted DNA, and 6.5 µl of nuclease-free water. The mixture was briefly vortexed and centrifuged. Amplification was carried out using a thermal cycler (Gene Atlas, Master cycler gradient, Model 482, Japan). After PCR, the amplified products were either analyzed by gel electrophoresis or stored at -20ºC for future experiments.

**Table 1:** Primers used in this study [11,12]

Genes	Primer name	Primer sequence (5'-3')	bp (base pair)
fosA	fosA-F fosA-R	ATCTGTGGGTCTGCCTGTCGT ATGCCCGCATAGGGCTTCT	271
fosA_3_	fosA3-F fosA3- R	CCTGGCATTTTATCAGCAGT CGGTTATCTTTCCATACCTCAG	221
fosA_4_	fosA4-F fosA4-R	CTGGCGTTTTATCAGCGGTT CTTCGCTGCGGTTGTCTTT	230
fosB	fosB-F fosB-R	CAGAGATATTTTAGGGGCTGACA CTCAATCTATCTTCTAAACTTCCTG	312
fosC	fosC-F fosC-R	CCTTGCTCACTGGGGATCTG TACAAGACCCGACGCACTTC	354
fosX	fosX-F fosX-R	TGTCCCTCACCTTCGACTCT TTGCTGGTCTGTGGATTTGC	382
FosL1	fosL1-F fosL1-R	GCAAGCGCAGACACAGACAG CTTGGCACAAGGTGGAACTTC	516
tetX4	tetX4-F tetX4-R	TTGGGACGAACGCTACAAAG CATCAACCCGCTGTTTACGC	181
tetA	tetA-F tetA-R	GTGAAACCCAACATACCCC GAAGGCAAGCAGGATGTAG	850

Agarose gel electrophoresis

PCR products were visualized using agarose gel electrophoresis on a 1.5% agarose gel prepared with 1X TBE buffer (Tris-EDTA). To prepare the gel, 0.18 g of agarose powder (LE, analytical grade, Promega, Madison, USA) was dissolved in 1.25 ml of TBE buffer, poured into a gel tray with a comb, and allowed to solidify. For loading, 5 µl of the PCR product was mixed with 1 µl of loading dye on parafilm and placed into the wells, while 2 µl of a 100 bp DNA ladder was combined with 1 µl of loading dye and loaded into a separate well. Electrophoresis was conducted at 230 V for 30 minutes. The gel was then stained with ethidium bromide (20 µl in 200 ml distilled water) and visualized under a UV transilluminator (Gel Doc, Major Science, Taiwan). DNA bands were sized by comparison to the 100 bp DNA ladder. For sequencing, the PCR-amplified bacterial DNA was purified using FAVOGEN DNA purification kits (Biotech Corp).

Statistical analysis

Statistical analysis was performed by Windows-based Statistical Package for Social Science (SPSS) software, version 22.0 (IBM Inc., Armonk, New York). Continuous data were expressed as mean, standard deviation, minimum, and maximum. Categorical data were summarized in terms of frequency counts and percentages. The sample size was based on the number of isolated bacteria during the sample collection period. 

Ethical consideration

All procedures of the present study were carried out in accordance with the principles for human investigations (i.e., Helsinki Declaration 2013) and also with the ethical guidelines of the institutional research ethics. Formal ethics approval was granted by the local ethics committee. Participants in the study were informed about the procedure and purpose of the study and the confidentiality of information provided. All participants consented willingly to be a part of the study during the data collection periods. All data were collected anonymously and were analyzed using the coding system.

## Results

**Figure 1:**
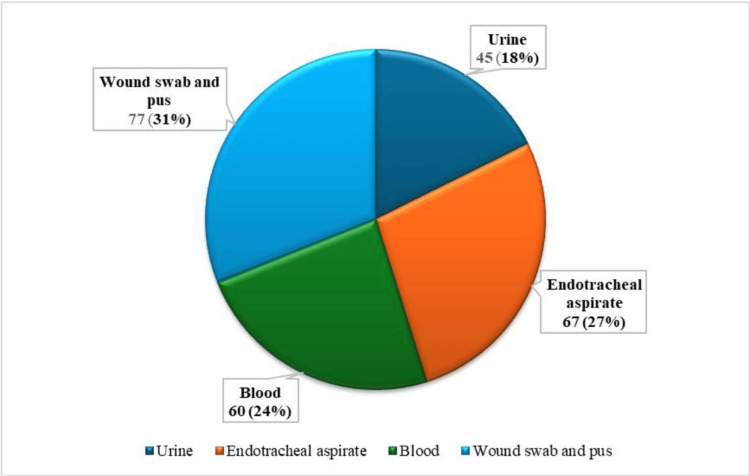
Culture positivity from different clinical samples

A total of 390 samples were analyzed in this study, among which 251 (64.4%) showed positive culture growth. The blood culture was positive in 60 (24.0%) cases. However, the urine culture was found in 45 (18.0%) cases. Endotracheal aspirate was positive in 67 (27.0%) cases. The positive culture was found in 77 (31.0%) cases in wound swab and pus culture (Figure 1).

**Figure 2:**
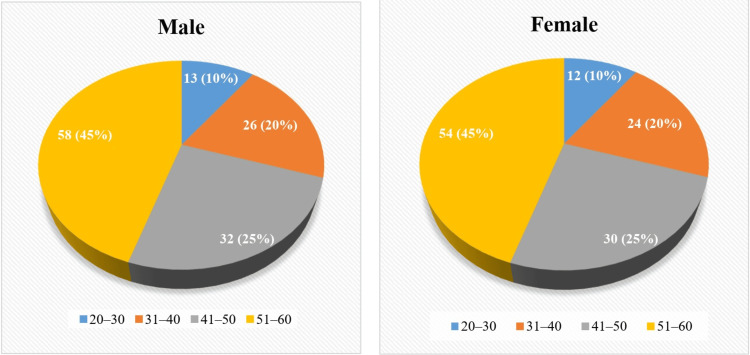
Age group distribution by gender

A comparative visualization of age distribution across genders, segmented into four distinct age brackets. The data highlights a significant contrast, with a higher concentration of females in the 20-30 age group and males in the 51-60 age group (Figure 2).

The distribution of Enterobacter species was recorded along with their multidrug-resistant (MDR), extensively drug-resistant (XDR), and pan drug-resistant (PDR) patterns, categorized by patient variables including age, sex, and clinical sample sources (Table 2).

**Table 2:** Distribution of Enterobacter Species, Resistance Patterns, and Patient Variables MDR - multidrug-resistant; XDR - extensively drug-resistant; PDR - pan drug-resistant

Sample source	Enterobacter species	MDR (%)	XDR (%)	PDR (%)
Urine	E. cloacae	15	5	2
Wound swab annd pus	E. cloacae	20	5	3
Endotracheal aspirate (ETA)	E. cloacae	10	3	3
Blood	E. cloacae	11.7	3.7	2
Urine	E. aerogenes	40.0	0.0	0.0

**Figure 3:**
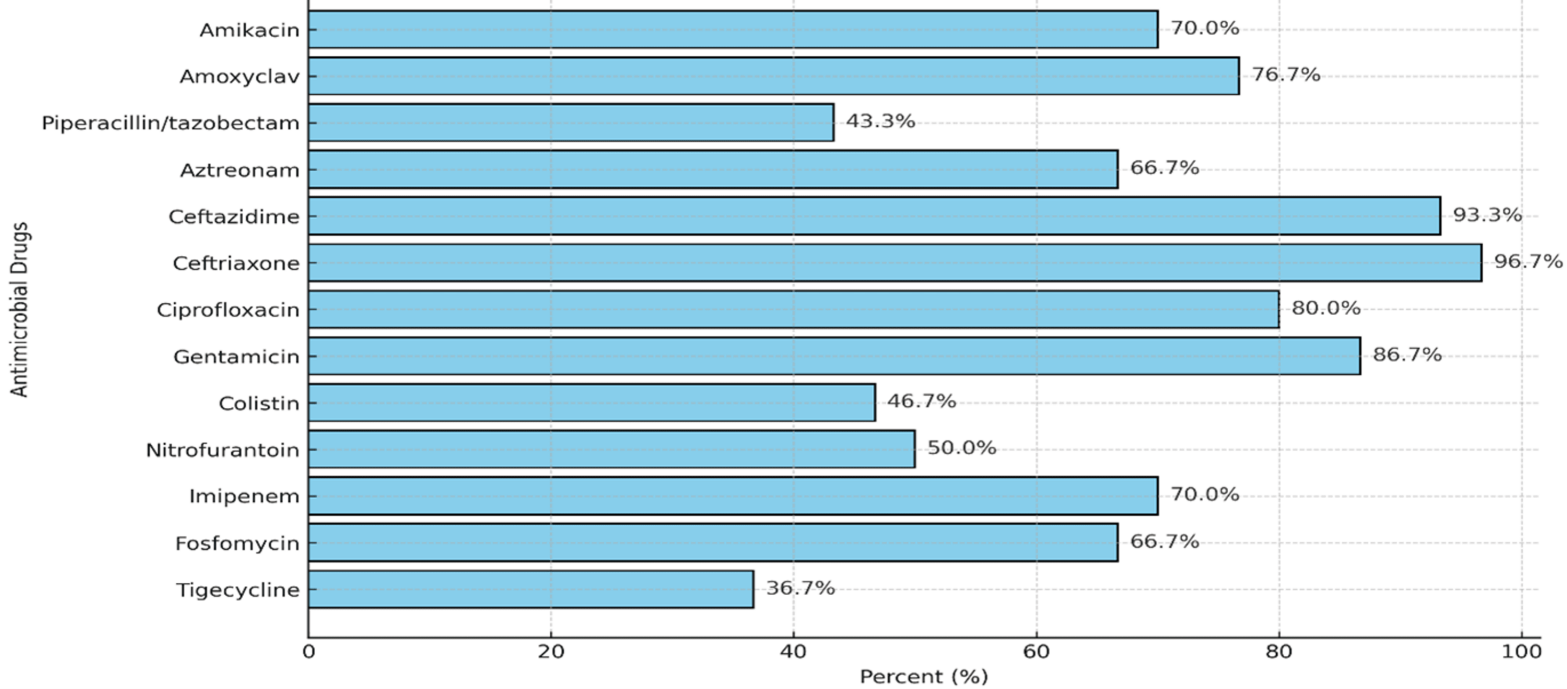
Antibiotic resistance pattern of isolated Enterobacter cloacae

The percentage of susceptibility to different antimicrobial drugs was recorded. Ceftriaxone (96.7%) and ceftazidime (93.3%) had the highest susceptibility, while tigecycline (36.7%) and piperacillin/tazobactam (43.3%) showed the lowest. Overall, β-lactam antibiotics demonstrated the greatest activity against the isolates (Figure 3).

**Figure 4:**
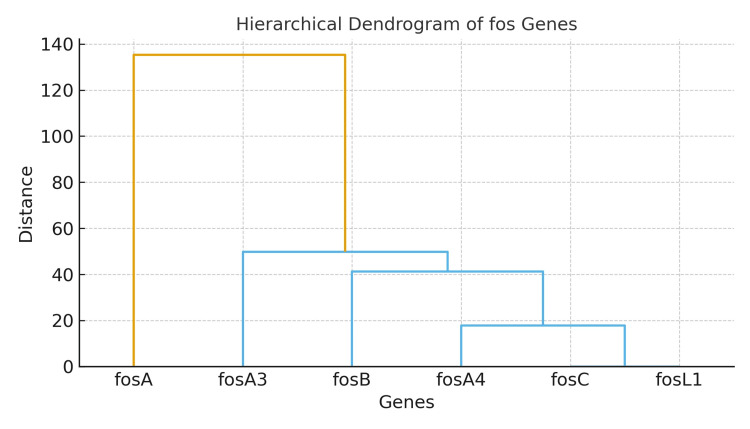
Hierarchical dendrogram showing clustering of fos genes based on percentage prevalence across clinical samples

The hierarchical cluster dendrogram below illustrates the clustering of fosfomycin resistance genes (*fos*A, *fos*A3, *fos*A4, *fos*B, *fos*C, and *fos*L1) according to their percentage distribution among different clinical samples. Genes with similar occurrence patterns, such as *fos*A and *fos*A3, are closely grouped, indicating possible co-occurrence or shared resistance mechanisms, while rarely detected genes like *fos*B, *fos*C, and *fos*L1 form separate branches (Figure 4).

Table 3 summarizes the prevalence of two tetracycline resistance genes, *tet*X and *tet*A, across 11 clinical samples from four sources.

**Table 3:** Prevalence of tetracycline resistance genes (tetX4, tetA) across sample type

Sample	Genes	
	*tet*X4	*tet*A
Urine (n=6)	0 (0.0%)	2 (33.3%)
Wound swab and pus (n=4)	0 (0.0%)	1 (25.0%)
Endotracheal aspirate (ETA) (n=1)	0 (0.0%)	0 (0.0%)
Blood (n=0)	0 (0.0%)	0 (0.0%)
Total (n=11)	0 (0.0%)	3 (27.3%)

## Discussion

This study presents novel insights into the genetic determinants of antibiotic resistance among *Enterobacter cloacae* isolates in Bangladesh. For the first time, the coexistence of *fos*A, *fos*A3, and *fos*A4 genes was detected, indicating diverse fosfomycin resistance mechanisms circulating in local clinical settings. The detection of the chromosomal *tet*A gene in 27.27% of isolates further suggests the role of efflux pumps in multidrug resistance. The co-occurrence of fos and tetA genes implies possible genetic linkage and co-selection under antibiotic pressure. Moreover, the absence of known tigecycline resistance genes highlights a gap in molecular surveillance, suggesting that tigecycline remains an effective treatment option in the region.

The global rise in antibiotic resistance represents a serious public health concern, contributing to significant morbidity and mortality [11]. Multidrug-resistant *Enterobacter *species are particularly concerning because of their role in nosocomial infections and their impact on therapeutic outcomes. In the present study, 251 out of 390 samples (64.36%) were culture positive, with 35 (13.94%) identified as *Enterobacter* species. This finding aligns with studies from Dhaka Medical College Hospital (DMCH), which reported culture positivity rates between 63.0% to 65.0% across different clinical specimens, including urine, wound swabs, blood, sputum, and pus [12-13]. Globally, *Enterobacter *accounts for approximately 11.0% of hospital-acquired infections involving the bloodstream, respiratory tract, and urinary tract [14].

Among the identified isolates, *Enterobacter cloacae* (85.7%) was the predominant species, followed by *Enterobacter aerogenes *(14.3%). This distribution corresponds with findings from previous studies in Bangladesh and India, which reported *Enterobacter cloacae* prevalence ranging from 77.0% to 79.0% isolates [15]. The predominance of *Enterobacter cloacae *may be linked to environmental persistence, antibiotic selection pressure, and similar infection control challenges shared across South Asian healthcare facilities.

Comparable findings have been reported in other Bangladeshi investigations, where *Enterobacter cloacae* accounted for more than 80.0% of *Enterobacter *infections and was strongly associated with ventilator-associated pneumonia and bloodstream infections [16]. International studies conducted in Iran, China, and other regions also describe *Enterobacter cloacae *as the most frequently isolated *Enterobacter* species, often carrying multidrug resistance genes and integrons. These observations support the global emergence of* Enterobacter cloacae *as an adaptable pathogen capable of acquiring and maintaining diverse resistance mechanisms.

The current study found a 70.0% imipenem resistance rate among *Enterobacter cloacae *isolates, similar to previous DMCH reports showing 70.97% resistance [16]. In contrast, lower rates have been reported in India (53.8%) and Palestine (12.2%), reflecting regional differences in antibiotic use and infection control practices [17-18]. The observed 66.7% fosfomycin resistance among isolates in this study was notably higher than the 36.0% resistance reported in Indian isolates [19].

Among fosfomycin-resistant isolates, 65.0% were positive for *fos*A, 20.0% for *fos*A3, and 15.0% for* fos*A4, demonstrating the predominance of *fos*A-mediated inactivation. This is consistent with earlier findings from DMCH, where *fos*A, *fos*A5, and *fos*B2 were reported among multidrug-resistant *Enterobacter *isolates [20]. In contrast, a study from China detected fosA3 in 26.1% of *Enterobacter cloacae* isolates [21], suggesting regional variability in resistance gene profiles likely influenced by differences in antimicrobial use and horizontal gene transfer events.

The presence of *tet*A genes in 27.3% of isolates indicates active efflux-mediated resistance, particularly via the major facilitator superfamily (MFS) mechanism. The coexistence of multiple resistance genes, such as *fos*A and *tet*A, raises concerns regarding plasmid-mediated gene transfer and the rapid dissemination of multidrug-resistant *Enterobacter* strains. The absence of known tigecycline resistance genes in local isolates is encouraging; however, continuous molecular surveillance for genes such as *tet*(X) and ramR mutations remains essential.

In summary, the findings of this study underscore the emergence of *Enterobacter cloacae *as a predominant multidrug-resistant pathogen in Bangladesh. The simultaneous presence of multiple resistance determinants reflects both local and global trends in antibiotic resistance evolution. Strengthening molecular surveillance, enforcing antibiotic stewardship, and improving infection control measures are vital to mitigating the further spread of resistance and preserving the efficacy of existing therapeutic options.

Limitations

The study was unable to detect certain resistance genes due to limitations in time and resources. Specifically, other tigecycline resistance genes, such as *tet*X6, were not analyzed. Similarly, some fosfomycin resistance genes, including *fos*L2, *fos*M1, and *fos*M2, could not be investigated. These constraints prevented a more comprehensive screening of all potential resistance determinants. Again, the small sample size, single-center design, lack of sequencing data, and absence of clinical outcome correlation are also limitations of the study.

## Conclusions

In conclusion, *Enterobacter cloaca*e shows a high rate of multidrug resistance, with the majority (around half) of isolates classified as MDR. There are several *fos *genes that are responsible for resistance to antibiotics.* *Among fosfomycin-resistant strains, the *fos*A gene was most common, while *fos*A3 and *fos*A4 appeared less frequently. This indicates the MDR profiles of the bacteria. In tigecycline-resistant isolates, the *tet*A gene was found at a lower level. This highlights the need for antimicrobial susceptibility testing before antibiotic use. However, it is very important to implement antibiotic stewardship to prevent the development of genes.
